# Longitudinal Alterations in the Control of Lateral Center of Mass Movement During Walking in a Patient With Unilateral Transtibial Amputation: A Case Study

**DOI:** 10.7759/cureus.61683

**Published:** 2024-06-04

**Authors:** Haruki Toda, Takashi Oshima, Takuya Ibara, Takaaki Chin

**Affiliations:** 1 Robot Rehabilitation Center, The Hyogo Institute of Assistive Technology, Kobe, JPN; 2 Orthopaedics, Hyogo Prefectural Rehabilitation Center, Kobe, JPN; 3 Functional Joint Anatomy, Graduate School of Medical and Dental Sciences, Tokyo Medical and Dental University, Tokyo, JPN; 4 Physical Medicine and Rehabilitation, Hyogo Prefectural Rehabilitation Center, Kobe, JPN

**Keywords:** unilateral transtibial amputation, uncontrolled manifold, segmental coordination, rehabilitation, prosthetic leg, gait, center of mass

## Abstract

This study assessed longitudinal changes in the control of the center of mass (CoM) in the lateral direction through gait reacquisition in an individual with unilateral transtibial amputation (UTTA). We examined a male patient with UTTA who could walk on a parallel bar. The marker trajectories and ground reaction forces were measured every two weeks (total: four times) using an optical motion capture system and force plates. After two measurements, the samples were collected without a parallel bar. Subsequently, we evaluated the CoM movement and its segmental coordination through uncontrolled manifold (UCM) analysis. After the second measurement, the walking speed and step length increased. The lateral CoM movements gradually increased toward the prosthetic side until the third measurement. In the fourth measurement, the CoM movement towards the prosthetic side was the smallest and closest to the intact side at the end of the stance phase. In addition, segmental coordination improved significantly. Enhanced gait performance delayed the improvement of segmental coordination for CoM movement in the lateral direction.

## Introduction

Individuals with unilateral transtibial amputation (UTTA) are often exposed to challenging gait rehabilitation. Only 30% of them can walk without using walking aids in their home environment, whereas more than 50% require a wheelchair [1]. In individuals with UTTA, reacquiring the ability to walk markedly improves their quality of life and has significant economic and social benefits [2]. We need to identify the key factors necessary for the reacquired gait process in these individuals.

Despite conserving knee joint function, individuals with UTTA continue to exhibit gait asymmetry [3]. Gait asymmetry explains the tendency to fall in the lateral direction [4]. Thus, gait asymmetries are associated with low gait-related performance [5] and induce musculoskeletal disorders such as low back pain in individuals with UTTA [6]. For individuals with UTTA, rehabilitation to improve gait asymmetry is necessary to walk comfortably in their daily lives.

To improve gait asymmetry, individuals with UTTA must adequately control the lateral movement of the center of mass (CoM). Gait with a transtibial prosthesis requires support by the foot without muscles or sensory information during the stance phase. To achieve this remarkable CoM control, the central nervous system has to control the coordination of whole-body segments for stabilizing body support during the stance phase.

The mechanism of how segments coordinate during lateral CoM movement while walking can be assessed through uncontrolled manifold (UCM) analysis [7-9]. A study on individuals with UTTA stated that, compared with the intact leg, the prosthetic legs demonstrated low intersegment coordination for producing propulsion [10]. However, longitudinal gait changes in lateral CoM movement control remain uninvestigated using UCM analysis in individuals with UTTA.

Generally, gait biomechanics studies of prostheses are conducted on people who have completed inpatient gait rehabilitation and can walk independently [11]. However, insight into how gait kinematics change during the gait reacquisition process is still unclear. Observing changes in lateral CoM movement and UCM indices during the gait reacquisition process for individuals with UTTA will help us understand how segmental coordination improves gait asymmetry. Therefore, our case study aimed to assess longitudinal changes in the control of lateral CoM movement throughout the gait reacquisition process in an individual with UTTA.

## Case presentation

Participant

**Figure 1:**
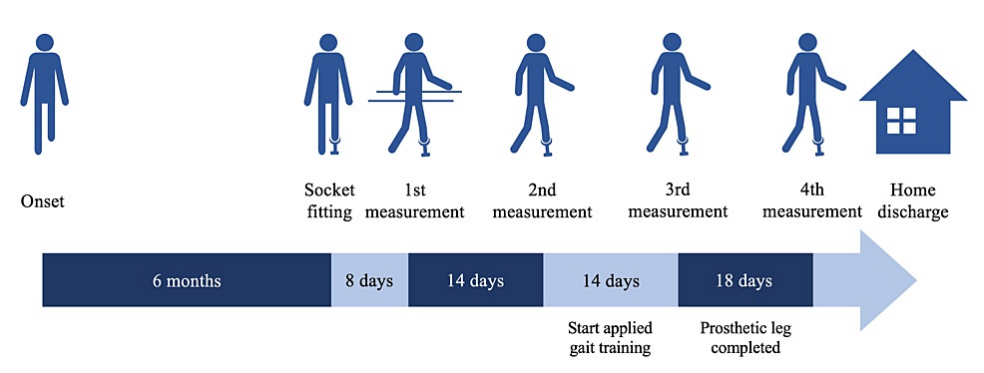
A timeline of the rehabilitation process of the participant in this study This image has been created by the authors.

This single-case longitudinal study included a male with a left UTTA caused by trauma. He was 49 years old, with a height of 1.79 m (179 cm, approximately 5 feet 10.47 inches) and a weight of 62.7 kg when wearing his prosthesis. The prosthesis included a Flex-Foot Assure (Össur, Reykjavik, Iceland) and a total surface bearing socket with a liner pin suspension system. Figure 1 illustrates the rehabilitation process. He had blisters on the transected skin grafts at the start of this study. Therefore, his prosthesis was continuously adjusted. However, his blisters healed completely before the final measurement. Consequently, his prosthesis adjustment was completed. His knee range of motion on the prosthetic side was 5° extension to 125° flexion. In the manual muscle test, his knee muscle strength on the prosthetic side was normal in extension and good in flexion. This study began eight days after gait rehabilitation initiation. Depending on his ability, he underwent 40 minutes of physical therapy at least once a day, six days a week. Walking was instructed by physical therapists to be safe and symmetrical. During hospitalization, he was allowed to perform strength and gait training voluntarily. Finally, he was discharged after the study.

Data collection

The participant walked along a linear path of 5 meters. To capture marker trajectories and measure ground reaction forces (GRF), we employed a system comprising eight infrared cameras (Vicon Vero, Vicon Motion Systems, Oxford, UK) and two floor-embedded force plates (Z13216 and Z13286, Kisler, Winterthur, Switzerland) with sampling frequencies of 100 Hz and 1,000 Hz. We attached 57 reflective infrared markers to the participant’s anatomical reference points. Measurements were taken every two weeks, for a total of four times. The participant walked into the parallel bars for the first time because his freehand gait was unstable. In this case, we instructed him not to touch the parallel bars except when he was off-balance. After the second measurement, the measurements were performed without the parallel bars.

Inverse kinematics

Joint kinematics was reconstructed using DhaibaWorks (https://www.dhaibaworks.com/) to align marker trajectories with individual body models. The models, with a link structure, were generated according to the participant’s marker positions during the T-pose and their body mass, including the prosthesis. DhaibaWorks computed the CoM locations and joint angles, including the neck, lumbothoracic, lumbopelvis, hip, knee, ankle, and segment angles, such as the head, thorax, lumbar, pelvis, thigh, and shank. Furthermore, inertial parameters were calculated from body weight based on able-bodied people because differences between prosthetic and healthy limbs are negligible [12]. Data were filtered through a fourth-order Butterworth low-pass filter with a 6 Hz cutoff frequency. Both walking velocity and stride length were calculated according to the anteroposterior shifts of the CoM movement. The segmental angles and mediolateral CoM displacements were isolated from a single-stance phase of the prosthetic leg extracted according to the GRF and then time-normalized, ranging from 0% to 100% over 10 trials. Step length was calculated from the anteroposterior CoM displacement during the stance phase. In addition, we plotted the CoM displacement according to the coordinates during the initial contact in the horizontal plane through the stance phase. We also calculated stance time from vertical GRF in the prosthetic leg. The walking speed was obtained from the step length and stance time. The CoM displacement, stance time, step length, and walking speed were averaged over 10 trials.

Uncontrolled manifold analysis

The influence of segment angle fluctuations (elemental variables) on mediolateral CoM coordination (performance variable) was investigated through UCM analysis using MATLAB R2023a (MathWorks Inc., Natick, MA). A spatial geometric model was developed by specifying the CoM coordinates in the frontal plane and articulating the angular relationships between various anatomical segments, namely, the shank, thigh, pelvis, lumbar region, thorax, and head, in congruence with a preexisting model delineated in our previous study [9]. The mathematical representation of the geometric model is as follows:

$COM_{ML}=l_{PS shank}\times d_{PS shank}\times M_{PS shank}\times cos\alpha _{PS shank}\times sin\Theta _{PS shank}+l_{PS thigh}\times d_{PS thigh}\times M_{PS thigh}\times cos\alpha _{PS thigh}\times sin\Theta _{PS thigh}+ l_{pelvis}\times d_{pelvis}\times M_{pelvis}\times cos\beta _{pelvis}\times cos\Theta _{pelvis}+l_{IS thigh}\times d_{IS thigh}\times M_{IS thigh}\times cos\alpha _{IS thigh}\times sin\Theta _{IS thigh}+l_{IS shank}\times d_{IS shank}\times M_{IS shank}\times cos\alpha _{IS shank}\times sin\Theta _{IS shank}+l_{lumbar}\times d_{lumbar}\times M_{lumbar}\times sin\Theta _{lumbar}+l_{thoracic}\times d_{thoracic}\times M_{thoracic}\times sin\Theta _{thoracic}+l_{head}\times d_{head}\times M_{head}\times sin\Theta _{head}$

In the given geometric model, *l* represents the length of individual segments, *d* signifies the CoM proportion within each segment, and *M* denotes the segmental mass on the prosthetic and intact sides. The angles theta (*θ)*, alpha (*α)*, and beta (*β)* correspond to the segmental angles on the sagittal, frontal, and transverse planes. The model comprises 13 elemental variables. A comparative evaluation against a three-dimensional whole-body model yielded a root mean squared error of 5.4 mm and a correlation coefficient of 0.90 for the mediolateral CoM position, even though the inertial parameters of the shank on the affected side were calculated from the participant’s body weight.

The geometric representation of the CoM displacement in the mediolateral direction was linearized using the Jacobian matrix (*J*). The zero space of *J* was calculated using the mean elementary variable. This subspace encapsulates the range of elemental variable perturbations that have no influence on the designated performance variable.

In the computational framework, the null space is characterized by *n* − *d* basis vectors, where *n* = 13 and *d* = 1 denote the dimensionality of the elemental and performance variables, respectively. Deviations in each trial’s elemental variable vector from the cross-trial mean elemental variable vector were time-slice specific and orthogonally projected onto this null space.

We determined the variance of uncontrolled manifold analysis (*V_UCM_*), which does not impact the CoM displacement, and the orthogonal variance (*V_ORT_*), which does impact the CoM displacement. The calculation methods for these variances follow those detailed in a previous study [9]. The total variance within the trials and the synergy index ($&Delta;𝑉$) were also computed based on established methods [9].

Given the non-normal distribution of $&Delta;𝑉$, Fisher’s z-transformation was applied to $&Delta;𝑉$ ($&Delta;𝑉$_Z_). *V_UCM_*, *V_ORT_*, and $&Delta;𝑉$_Z_ were averaged over the single-stance phase, and their changes over time due to improvements in walking ability were subsequently analyzed.

Assessment of gait reacquisition

**Figure 2:**
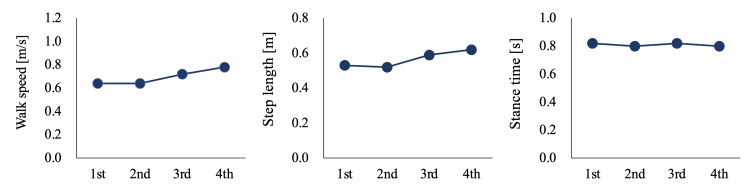
Spatiotemporal parameters for each measurement

Figure 2 shows the longitudinal changes in gait performance. The walking speed and step length increased from the second to the fourth measurements. The stance time did not significantly change through the measurements.

**Figure 3:**
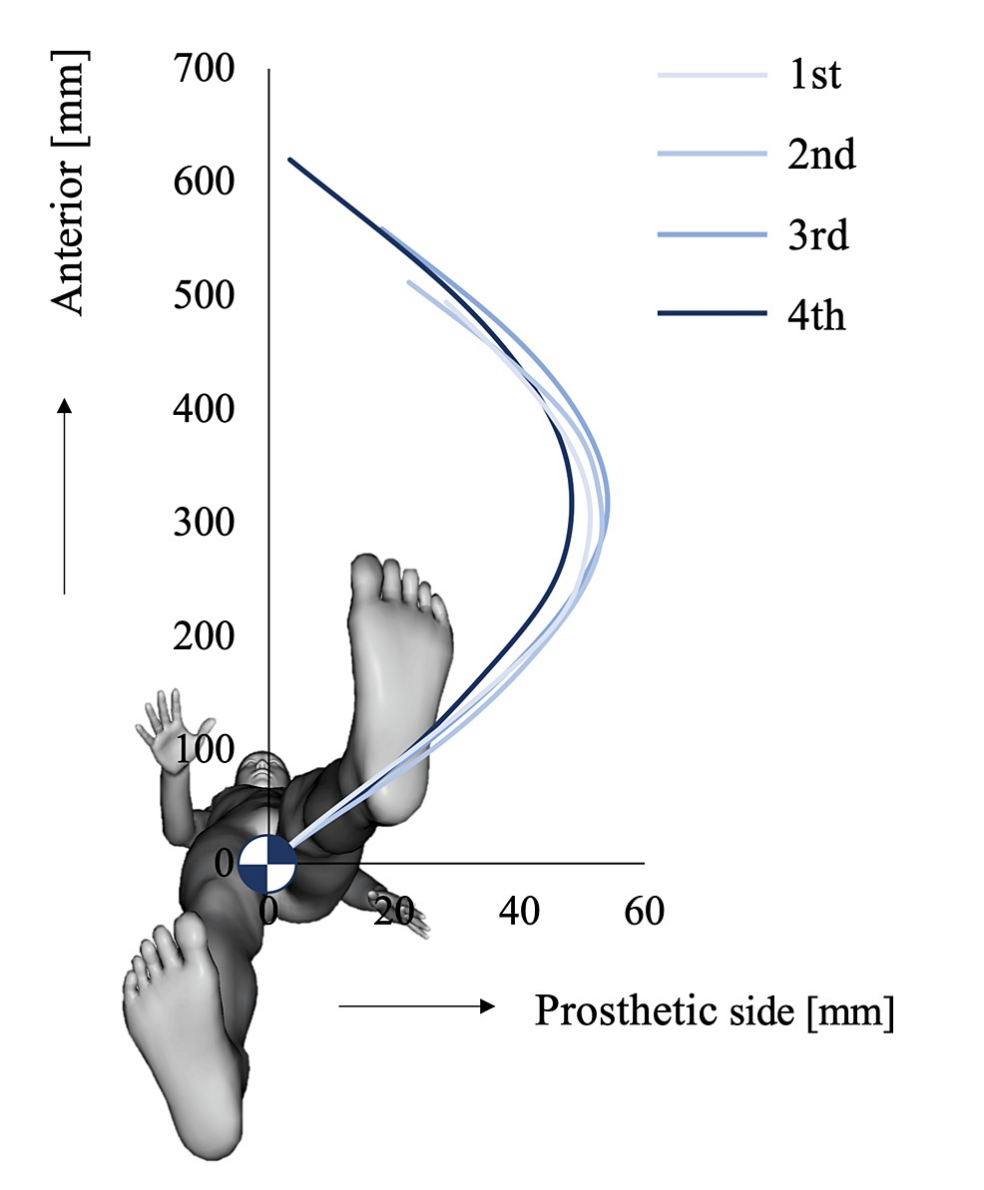
The center of mass (CoM) movement is plotted on the horizontal plane for each measurement. The CoM positions were corrected so that the position at the initial contact on the prosthetic side is at the origin. This figure is the original work of the authors.

Figure 3 indicates a longitudinal plot of the CoM movement in the horizontal plane during the stance phase of the prosthetic leg. During such a phase, the amount of lateral CoM movement to the prosthetic side gradually increased from the first to the third measurement, but it was the smallest on the fourth measurement. At the end of the stance phase, the lateral CoM movement to the intact side increased with each measurement.

**Figure 4:**
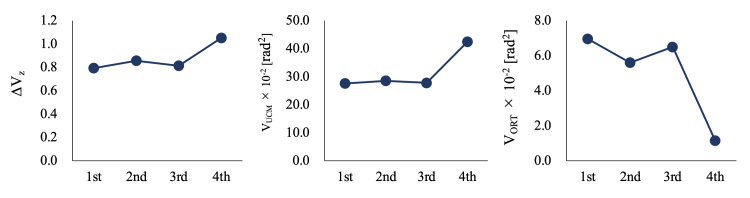
Uncontrolled manifold (UCM) indices for each measurement *Δ𝑉_Z_*: Fisher’s z-transformation applied to the synergy index; *V_UCM_*:*_ _*variance of uncontrolled manifold analysis; *V_ORT_*: orthogonal variance

Figure 4 presents the longitudinal graphs of UCM indices. Both *V_UCM_* and *V_ORT_* did not change until the third measurement. On the fourth measurement, *V_UCM_* increased while *V_ORT_* decreased; consequently, *ΔV_Z_* was significantly increased.

## Discussion

We examined longitudinal alterations in lateral CoM movement and the segmental coordination to control it during the stance of gait in individuals with UTTA. When segmental coordination significantly improved before home discharge, the CoM displacement to the prosthesis side decreased. This case study is the first to suggest a change in the strategy of controlling the CoM movement after the walking ability is improved during the gait reacquisition process of a patient with UTTA.

The CoM movement to the prosthetic side increased until the third measurement. Individuals with UTTA generally have difficulty shifting weight to the prosthetic leg during the sit-to-stand motion [13] and walking [14]. Therefore, in the rehabilitation setting, they were instructed to load on the prosthetic leg. However, in able-bodied individuals, the CoM movement to the stance phase of walking in the lateral direction is approximately 2 cm, and the CoM moves without passing on the foot during walking [15]. The overweighting of the prosthetic leg might be too conscious, resulting in no change in segmental coordination. On the fourth measurement, the CoM movement to the prosthetic side was reduced, while the segmental coordination was increased. The optimized prosthetic leg before this measurement may have benefited from applied gait training that started after the second measurement. However, we cannot explain why the lateral control of the CoM during walking changed at this time in this case. Therefore, further research is needed to clarify the relationship between segmental coordination during walking and physical status, function, and prosthetic adjustment and the effect of applied gait training on segmental coordination during walking.

Improved segmental coordination for lateral CoM movement is needed to facilitate the reacquisition of gait in individuals with UTTA. In healthy people, segmental coordination for lateral CoM movement is altered by vibrotactile stimulation [9]. Recently, vibrotactile stimulation was used in people with transfemoral amputees to improve the gait asymmetry of spatiotemporal parameters [16]. Sensory stimulus may be an intervention option to facilitate segmental coordination improvement.

The segmental coordination did not change until the third measurement. In the first to second measurements, the participant could walk without parallel bars, but his gait was unstable. Therefore, gait performance remained unchanged. However, on the third measurement, despite prolonged step length and increased gait speed, segmental coordination did not improve. This result suggests that improvements in gait performance and segmental coordination are separate. The walking speed and step length of individuals with UTTA are related to hip and ankle kinetics in intact legs [17]. Hence, compensation for the intact leg may have improved gait performance. Consequently, segmental coordination during walking improves with a delay in performance.

This study has several limitations. From the current study results, we cannot explain the causal relationship between lateral CoM movement and segmental coordination. In addition, we did not evaluate changes in the participant’s physical function. Lateral control during walking was related to physical function and activity [18]. Thus, we cannot explain the change in the lateral CoM movement and the improvement of segmental coordination on the fourth measurement.

## Conclusions

This case study showed the longitudinal changes in lateral CoM movement and the segmental coordination required to control this movement in the gait reacquisition process of an individual with UTTA. Segmental coordination for lateral CoM movement improved after the onset of an increase in gait performance. Further studies are needed to identify the physical function required to improve the segmental coordination for CoM movement in the lateral direction.
